# Evaluation of the Antiwrinkle Activity of Enriched Isatidis Folium Extract and an HPLC–UV Method for the Quality Control of Its Cream Products

**DOI:** 10.3390/plants9111586

**Published:** 2020-11-16

**Authors:** Dan Gao, Chong Woon Cho, Cheong Taek Kim, Won Seok Jeong, Jong Seong Kang

**Affiliations:** 1 College of Pharmacy, Chungnam National University, Daejeon 34134, Korea; gaodan521361@hotmail.com (D.G.); chongw113@naver.com (C.W.C.); 2 RNS Inc., Daejeon 34014, Korea; happilion@biorns.com (C.T.K.); zmal1329@biorns.com (W.S.J.)

**Keywords:** isatidis folium, HPLC-PDA-ESI-MS/MS, antiwrinkle activity, quality control, response surface methodology

## Abstract

Currently, many extracts from natural sources are added to cosmetic products for reducing facial aging and wrinkles. This study investigated the antiwrinkle activity of enriched extract of Isatidis Folium used for a novel antiwrinkle cream product. The result demonstrated that this enriched extract has excellent antiwrinkle activity by significantly inhibiting mRNA expression of matrix metalloproteinase-1, matrix metalloproteinase-3, and pro-inflammatory cytokines IL-1β and upregulating the mRNA expression of IL-4 and procollagen. Additionally, to implement effective quality control of the entire manufacturing process of antiwrinkle cream products based on the enriched extract of Isatidis Folium, the main chemical constituents of the enriched extract of Isatidis Folium was evaluated by high–performance liquid chromatography-photodiode array-tandem mass spectrometry (HPLC-PDA-ESI-MS/MS), five constituents were undisputedly confirmed. An HPLC-UV method in 15-min analysis time for quality assessment of the entire manufacturing process of antiwrinkle cream products was proposed and validated. The optimal conditions for extracting TMCA (3,4,5-trimethoxycinnamic acid) from the developed antiwrinkle cream products were determined using response surface methodology based on central composite design. The established HPLC method and optimal extract condition are suitable for routinely analyzing this novel antiwrinkle cream product.

## 1. Introduction

The wrinkle formation, which is the most obvious skin-aging sign, is affected by UV irradiation, exterior environment, and age [1,2]. Notably, deficiency in collagen synthesis and matrix metalloproteinase (MMP) overexpression are the main causes of wrinkles [3,4]. Collagen comprises approximately 75% of the dry weight of skin and is critically involved in the strength and structural elasticity of skin tissue [5]. Procollagen is the main raw material for collagen synthesis [6]. MMPs are enzymes that directly degrade a large number of extracellular matrix components, including collagen, elastin, and gelatin [7]. UV radiation contributes to MMP overexpression, such as MMP-1 (interstitial collagenase), which inaugurates the degradation of type I and III collagens, and MMP-3 (stromelysin 1), which necessitates the degradation of type IV and activation of pro-MMP-1 [8,9]. However, UV exposure has been suggested to induce inflammatory responses and photoaging in the skin, which stimulates the transcription of proinflammatory cytokine genes, such as IL-1β, IL-6, and IL-8 [10,11,12]. Therefore, evaluating specific materials for promoting procollagen synthesis and inhibitory of MMP expression could be used to screen potential compounds or extracts used in producing the antiwrinkle products.

Isatidis Folium is the dried leaves of *Isatia indigotica* Fort. Belonging to the Cruciferae family and is a well-known herb with various biological activities and is deemed a promising agent for treating skin diseases because of its rich anti-inflammation and antioxidant components [13,14,15]. Isatidis Folium demonstrated the best antiwrinkle effect among the prescreened plants. Consequently, using Isatidis Folium extracts as a raw material in developing effective antiwrinkle cosmetics is a promising approach in the cosmetic industry innovation.

Some previously developed analytical methods to control the Isatidis Folium quality are thin-layer chromatography, high-performance liquid chromatography-ultraviolet (HPLC-UV), HPLC-atmospheric pressure chemical ionization (APCI)-mass spectrometry (MS), HPLC-electrospray ionization (ESI)-MS, nuclear magnetic resonance, and Fourier Transform (FT)-Raman spectroscopy [16,17,18,19]. However, none of these established methods can be used to identify and quantify the bioactive compounds in our enriched Isatidis Folium extract because of many components with a high amount in the crude extract (such as alkaloids and glycosides) were removed during the purification process. Unfortunately, it is necessary to profile the chemical constituents of enriched Isatidis Folium extract and find characteristic compounds to ensure the quality and safety of the novel antiwrinkle cream products.

Therefore, this study investigated the antiwrinkle activities of Isatidis Folium by evaluating its potency in regulating MMP-1, MMP-3, procollagen, and proinflammatory cytokine gene expression using the technique of real-time reverse-transcription polymerase chain reaction (real-time RT-PCR). A new antiwrinkle cream product was developed using enriched extracts of Isatidis Folium that exhibited excellent antiwrinkle activity. We investigated the major chemical constituents of enriched Isatidis Folium extracts using HPLC-photodiode array (PDA)-tandem mass spectrometry (HPLC-PDA-MS/MS) and established an effective and rapid analytical method that allowed us to determine characteristic compound TMCA (3,4,5-trimethoxycinnamic acid) in all steps of this product production process.

## 2. Results and Discussion

### 2.1. Antiwrinkle Activity

**Figure 1:**
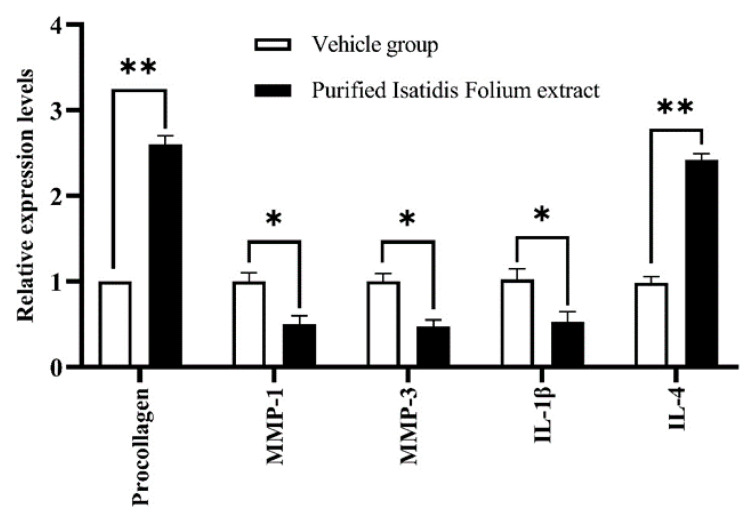
The effect of enriched Isatidis Folium on mRNA expression of procollagen, MMP-1, MMP-3, IL-1β, and IL-4. (* *p* < 0.05), (** *p* < 0.01) versus vehicle-treated group.

The mRNA expression of MMP-1, MMP-3, procollagen, IL-1β, and IL-4 involved in wrinkle formation was evaluated using RT-RCR in CCD-986sk cells treated with enriched Isatidis Folium extract at 5-μg/mL concentration. Measurements proving the enriched Isatidis Folium extract effects on mRNA expression of procollagen and IL-4 in vitro showed an increase in mRNA expression of IL-4 and procollagen 2.5 and 2.4 times, respectively, compared with the vehicle group (Figure 1). Meanwhile, Figure 1 illustrates that the enriched Isatidis Folium extract can downregulate the mRNA expression of MMP-1, MMP-3, and pro-inflammatory cytokines IL-1β. These results revealed that the enriched Isatidis Folium extract has an excellent antiwrinkle efficacy, suggesting that it could be used to develop antiwrinkle cream products.

### 2.2. Structural Characterization of the Main Compounds from Enriched Isatidis Folium Extract

**Figure 2:**
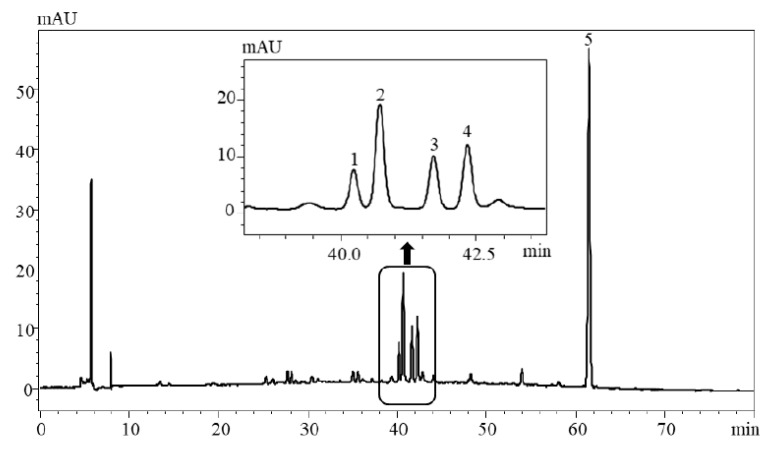
High–performance liquid chromatography-photodiode array (HPLC-PDA) chromatogram of detected compounds in enriched Isatidis Folium extract. HPLC conditions: column; Optimapark C_18_ (4.6 mm × 250 mm, 5 μm), elution; 0.1% formic acid aqueous solution (A) and acetonitrile-formic acid (99.9:0.1, B) at a linear-gradient elution program from 5% to 70% for 80 min, column temp; 30 °C, detection wavelength; 360 nm.

**Figure 3:**
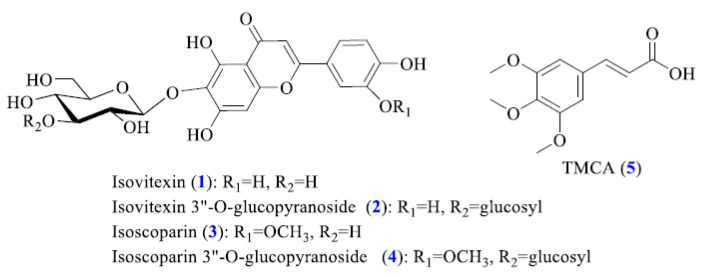
Chemical structures of identified compounds from enriched Isatidis Folium extract.

HPLC-PDA-ESI-MS/MS was adopted to tentatively identify major compounds in enriched Isatidis Folium extract. While the PDA data were effective for identifying the class of the flavonoid and phenolic acid compounds, the MS data supplied further structural characterization. The produced fragmentation of the predominant negative ions in the MS/MS mode were used to produce fragmentation ions to obtain more detailed information about the molecular masses of various compounds [20,21]. Compound identification was confirmed by comparing their UV spectra, MS spectra (Figures S1–S5, Table 1), and elution order with those in the previously reported literature of components found in Isatidis Folium. Figure 2 shows the HPLC-PDA chromatogram monitored at 360 nm profiles of enriched Isatidis Folium extract. First, peak 5 was clearly confirmed to be TMCA by comparing their maximum UV wavelength, MS spectra and retention time with the reference compound (Figure 3). TMCA was deemed as an active ingredient in Isatidis Folium, which was suggested to have anti-inflammation, antitumor, and anticonvulsant activities [22,23,24]. Therefore, as the most representative component in the enriched extract, TMCA can be selected as a marker compound to evaluate this enriched extract quality. Moreover, all UV spectra with their λmax at approximately 270 and 330 nm of peaks 1–4 agree with flavonoid characterization [25]. Based on the ESI-MS/MS and literature data, peaks 1–4 were assigned as isovitexin, isovitexin-3″-O-glucopyranoside, isoscoparin, and isoscoparin-3″-O-glucopyranoside (Figure 3) [15,16].

### 2.3. Optimizing the Analytical Method

**Figure 4:**
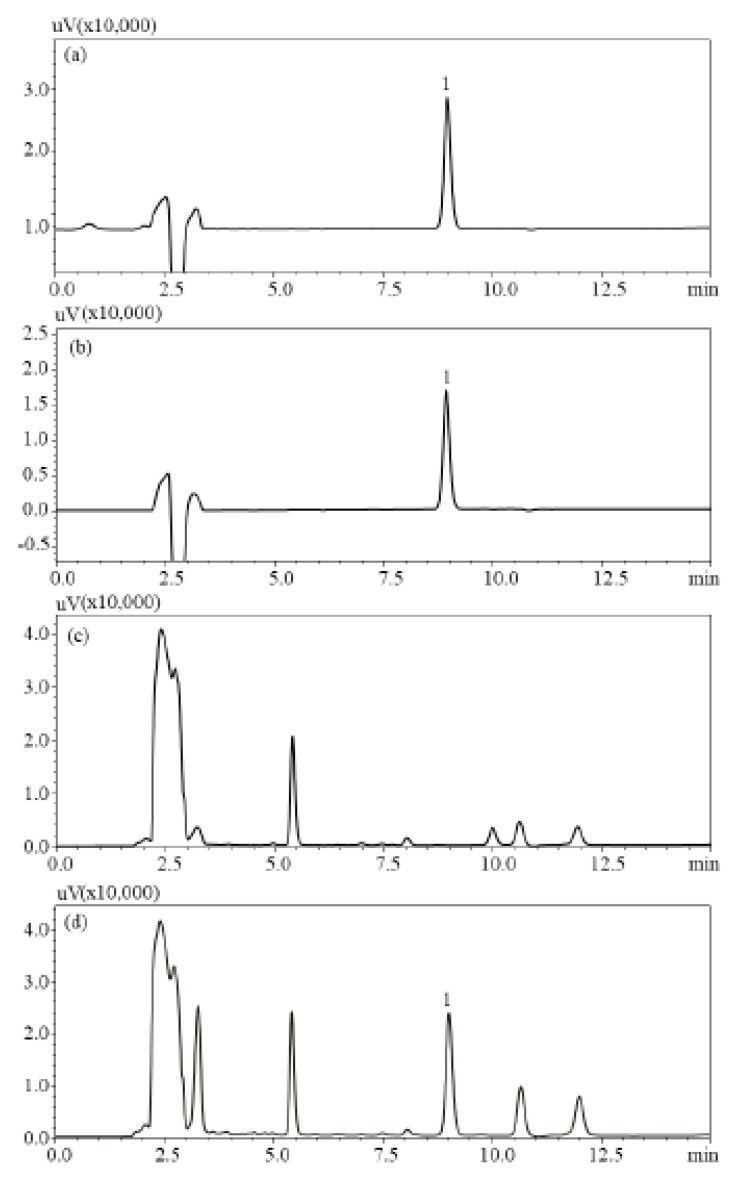
High–performance liquid chromatography-photodiode array (HPLC-PDA) chromatograms of (**a**) the standard solution of TMCA, (**b**) the powder of enriched Isatidis Folium extract, (**c**) cream matrix, and (**d**) developed anti-wrinkle cream products. HPLC conditions: column; Optimapark C_18_ (4.6 mm × 250 mm, 5 μm), mobile phase; 0.1% formic acid aqueous solution/acetonitrile (65:35), column temp; 30 °C, detection wavelength; 302 nm Peak 1. 3,4,5-trimethoxycinnamic acid, Retention time: 8.94 min.

To obtain rapid and simple chromatographic conditions, various HPLC parameters were compared and explored, including the mobile phase compositions (acetonitrile-water and methanol-water containing different buffers, such as formic acid, acetic acid, and trifluoroacetic acid), the temperature of column (25, 30, 35, and 40 °C), and the flow rate of the mobile phase (0.7, 0.9, 1.0, and 1.2 mL/min). Using the PDA for obtaining a satisfying resolution and separation, the UV spectra of TMCA were characterized. The optimal wavelength was set up at 302 nm. Certainly, a good peak shape, separation, and resolution of TMCA were obtained under the optimized conditions. HPLC chromatograms of (a) the standard solution of TMCA, (b) enriched powder of Isatidis Folium extract, (c) cream matrix, and (d) developed antiwrinkle cream products are shown in Figure 4. The retention time of TMCA was 8.94 min and no interference was observed in the chromatograms of the samples, which showed that this proposed HPLC analytical conditions would be useful and powerful for routinely determining TMCA in the production process of antiwrinkle cream products.

### 2.4. Method Validation

The proposed method was validated in terms of linearity, limit of quantitation (LOQ), limit of detection (LOD), accuracy, precision, repeatability, and recovery test. The results showed that this developed method was reliable, stable, and conducive for determining TMCA in our developed antiwrinkle cream products (Table S1).

#### 2.4.1. Linearity, LOD, and LOQ

The linearity of the peak area versus concentration of TMCA was shown by building up a calibration curve with five concentrations under optimized analytical conditions. The linearity range of TMCA was 1.0–10.0 μg/mL. The regression equation between the peak area (Y) and the concentration (X) of TMCA was Y = 360X − 60. The correlation coefficient was 0.9996 for TMCA. The LOD and LOQ were calculated by setting up the detector response signal to noise ratio of 3:1 and 10:1, respectively. The values of LOD and LOQ were 6.08 ng/mL and 18.44 ng/mL, respectively, which reflected the high sensitivity of this novel HPLC method.

#### 2.4.2. Accuracy and Precision

Accuracy and precision were obtained by analyzing three concentrations (low, middle, and high) of the TMCA standard five times, which were expressed as the relative standard deviation (RSD). The precision of intra-day and inter-day for the TMCA was 0.2–0.6% and 1.9–3.2%, respectively, and the accuracy of this analytical method ranged from 97.3–107.0% (Table S1).

#### 2.4.3. Repeatability

Repeatability was used to verify the stability of the HPLC instrument after consecutive injection. In this study, repeatability was obtained by calculating the retention times and contents of TMCA in cream samples. The RSD of retention time and concentration of TMCA were 0.23% and 0.18%, respectively. The analytical method was shown to be effective and accurate.

#### 2.4.4. Recoveries

The recoveries were obtained by calculating the ratio percentage as 100× (found concentration–original concentration)/spiked concentration. A certain concentration of TMCA in three concentrations (5, 6, and 7 μg/mL) was added to the cream solutions, extracted, and determined using the developed method. Acceptable recoveries of this analytical method are showing in Table S1.

### 2.5. Optimization of Sample Preparation for Extracting TMCA in Developed Antiwrinkle Cream Products

From the results of single-factor experiments, the extraction temperature had no significant effect on TMCA extraction (*p* > 0.05), and the two main empirical parameters of sonication time (A) and methanol-to-material ratio (B) were further optimized using response surface methodology (RSM) by central composite design (CCD). RSM is an all-powerful approach to optimize different extract conditions for obtaining the best response values [26,27]. Thirteen designed experiments were accomplished and the response values (the content of TMCA) are listed in Table 2. A quadratic model was suggested to predict the response values. The response to the content of TMCA (Y) was given by the following Equation (1):

Y = 29.63 − 1.24 A − 0.917 B + 1.10 AB − 4.23A^2^ − 3.09 B^2^, (1)

To evaluate the statistically significant difference in this model, Fisher’s test and *p*-value were conducted. The model *F*-value of 175.99 indicated that this mode is significant [28]. The *p*-value < 0.05 indicated that the model terms were significant. Analysis of variance (ANOVA) was performed to analyze the significance of the two independent variables and interaction terms, and the results implied that the model terms of A, B, AB, A^2^, and B^2^ were significant (Table S2). Conclusively, the TMCA extraction efficiency was greatly affected by sonication time and methanol-to-material ratio. Furthermore, a lack-of-fit *p*-value of 0.1297 indicated that the lack-of-fit was not significantly related to pure error [29].

The coefficient of determination (*R*^2^ and adjusted-*R*^2^) was used to evaluate the model fitting quality. The predicted *R*^2^ of 0.9495 properly agreed with the adjusted *R*^2^ of 0.9865, and the difference was < 0.2. Adeq Precision of 32.296 indicated an adequate signal. The coefficient of variation (CV) was selected to assess the predicted model reproducibility, which was obtained by calculating the ratio of the standard error to the mean value [30]. The CV of 1.52% suggested that the predicted model has great reproducibility. In this way, the model could be used to navigate the design space. From Figure S6a, the predicted and actual data plots were distributed near the line, which revealed that the proposed model was excellently fitted. Figure S6b shows that the residual plots were randomly scattered, which demonstrated that the variance of the experimental results was consistent for all response values [26,31].

**Figure 5:**
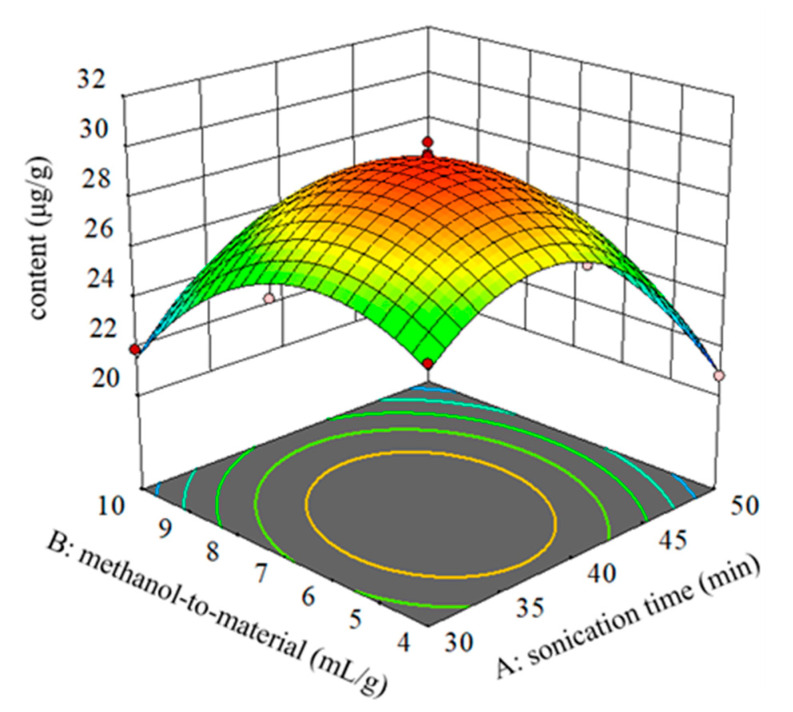
Response surface (3-D) plot showing the effect of sonication time (min) and methanol-to-material (mL/g) on the extraction yields of TMCA (3,4,5-trimethoxycinnamic acid). (**A**) sonication time (min); (**B**) methanol-to-material (mL/g).

A three-dimensional (3D) response surface was used to show the impact of each variable on the response value. Figure 5 illustrates the interactive effects of sonication time and methanol-to-material ratio on the yield of TMCA. The optimal extract conditions predicted by this excellent model are shown as follows: sonication time of 38.31 min and the methanol-to-material ratio of 6.47. To confirm the adequacy and validity of the established regression models, three parallel validation experiments were conducted under predicted conditions and the results are listed in Table S3. The matching degree between the predicted and real experimental results was 97.85%, indicating that the RSM was a reliable approach to obtain the optimum extract conditions of the TMCA from the novel antiwrinkle cream products.

### 2.6. Method Application to Raw Materials and Products

To evaluate the reliability of the novel HPLC method, different batches of enriched Isatidis Folium extract powder and developed antiwrinkle cream products were analyzed with the proposed method. The analyte contents were calculated using the standard calibration curves and the results are summarized in Table 3. No significant differences in TMCA content among different batches of samples (*p* > 0.05) were observed, indicating that the quality of the enriched Isatidis Folium extract and developed antiwrinkle cream products were even and stable, and the proposed method is practical for the whole industrial process for producing the antiwrinkle cream products containing the enriched Isatidis Folium extract.

## 3. Materials and Methods

### 3.1. Plant Material and Reagents

The authenticated raw material samples of Isatidis Folium were purchased from the company of Hanyajaesijang (Seoul, Korea), which was produced in July 2019 from China. The reference compound of TMCA (99%) was obtained from Alfa Aesar (Alfa Aesar, Ward Hill, MA, USA). Cream matrix and novel antiwrinkle cream products were provided by RNS Inc. (Daejeon, Korea).

HPLC-grade acetonitrile and methanol were obtained from Burdick & Jackson (Muskegon, MI, USA); a buffer containing formic acid, acetic acid, and trifluoroacetic acid (MS grade) was purchased from Sigma-Aldrich (St. Louis, MO, USA). Distilled water was enriched using a Milli-Q system (Sinhan, Seoul, Korea). The primers used in this study were purchased from Bioneer Inc. (Daejeon, Korea).

### 3.2. Preparing Enriched Isatidis Folium Extract and Cosmetic Creams

Isatidis Folium powder (100 g) was mixed with cyclohexane (500 mL) for removing the fat-soluble compounds and soaked at room temperature for 72 h. Then, the cyclohexane extract solution was filtered with a filter paper, and residues were dried and re-extracted with methanol (500 mL) by soaking for 72 h at room temperature. Subsequently, to obtain the powder, the methanol extract was further filtered and concentrated. The extract powder was mixed 5 times with ethyl acetate solution and stirred for 24 h. The ethyl acetate extract solution was then filtered and the residue was dissolved 5 times in methanol, after which the powder of the enriched Isatidis Folium extract was obtained by vacuum concentration. Finally, the enriched Isatidis Folium extract (1 g) was added to 1,3-butylene glycol (99 mL) for making the solution that would be used in antiwrinkle cream products. The obtained 1,3-butylene glycol solution (2.5 g) was added to the cream matrix (97.5 g) for manufacturing novel antiwrinkle cream products.

### 3.3. Preparation of Sample and Standard Solution

The enriched Isatidis Folium extract powder (0.1 g) was dissolved in 10-mL methanol and then diluted 200 times for HPLC analysis. The developed antiwrinkle cream (2 g) was dissolved in 4-mL methanol and sonicated for 40 min at 50 °C. Furthermore, a standard stock solution (10 mg/mL) of TMCA was prepared by dissolving it in methanol.

All stock and working solutions were stored in a refrigerator at 4 °C until use. All samples and standard solutions were filtered using a 0.22-μm membrane filter before HPLC analysis.

### 3.4. Optimizing Experimental Parameters

The maximum yield of target compounds is crucial for pharmacological testing and production. In this study, the “one-factor-at-a-time” methodology was used to identify an acceptable level for evaluated factors. First, different extraction times (0, 10, 30, 40, 50, and 60 min) were investigated using methanol as the solvent and a solvent-to-material ratio of 4:1. Then, extraction temperatures (30 °C, 45 °C, 60 °C, 75 °C, and 80 °C) were examined using methanol as the solvent and a solvent-to-material ratio of 4:1 for 40 min. Finally, the methanol-to-material (2, 4, 7, 10, 16, and 22 mL/g) for extracting TMCA was determined using 40-min extraction time and 30 °C extraction temperature. The TMCA in novel antiwrinkle cream products (μg/g) were used as response values to establish a proper range among the studied factors. The results of these independent factors were optimized by RSM using Design-Expert software (version 12, Sta-Ease Inc., Minneapolis, MN, USA).

### 3.5. HPLC-PDA-ESI-MS/MS Analysis

HPLC-PDA-ESI-MS/MS analysis was performed using a ProminenceTM HPLC system (Shimadzu, Kyoto, Japan) coupled to an LCMS-8040 system equipped with an electrospray ion source (ESI) (Shimadzu, Kyoto, Japan). A linear-gradient elution program from 5% to 70% for 80 min with a flow rate of 0.5 mL/min was run for separation. The wavelength for PDA detection was set up between 200 and 400 nm. The mass spectrometer was performed in negative and positive modes, and the interface voltage was set up at −3.5 kV and 3.5 kV, respectively. Other analytical parameters were displayed as follows: a nebulizing gas flow rate = 3 L/min, a desolvation line temperature = 250 °C, a drying gas flow = 15 L/min, and a heat block temperature = 400 °C. The MS^2^ data were obtained using a production survey scan in positive and negative modes.

### 3.6. Quality Control of Enriched Isatidis Folium Extract and Developed Antiwrinkle Cream Products

The HPLC analysis was performed on a Shimadzu HPLC system (Kyoto, Japan) equipped with a PDA detector. All samples were eluted through an Optimapark C_18_ column (4.6 mm × 250 mm, 5 μm) at a column temperature of 30 °C and a flow rate of 1 mL/min. The mobile phase comprising 0.1% formic acid in aqueous solution (solvent A) and acetonitrile (solvent B) at a ratio of 65:35 was used in the isocratic mode. PDA detection was used to conduct spectral scans over the range 200–400 nm, and detection wavelengths were monitored at 302 nm for determining and quantifying TMCA. Additionally, different validation parameters of this developed HPLC analytical method, such as precision, accuracy, LOQ, LOD, repeatability and others were assessed according to the International Council for Harmonization Guidance for Industry and Method Validation Guidelines of the Korean Ministry of Food and Drug Safety [32].

### 3.7. Cell Culture 

Human fibroblast CCD-986sk cells were purchased from the Korean Cell Line Bank (KCLB, Seoul, Korea). CCD-986sk cells were cultured and maintained in Iscove’s Modified Dulbecco’s Medium (Hyclone Co., Logan, UT, USA) complemented with 10% FBS (Hyclone Co., Logan, UT, USA) and 1% antibiotic antimycotic (100 U/mL penicillin and 100 μg/mL streptomycin) at 37 °C, 5% CO_2_ incubator.

### 3.8. Real-Time Reverse-Transcription Polymerase Chain Reaction (Real-Time RT-PCR)

CCD-986sk cells were added to a 6-well plate at a concentration of 5 × 10^4^/well and then cultured in a 5% CO_2_ incubator at 37 °C. After that, the cells were treated with the enriched Isatidis Folium extract (5 μg/mL) for 24 h. According to the manufacturer’s instructions, total RNA was isolated from CCD-986sk cells with TRIzol Reagent (Cat. No: 15596018, Life Technologies, New York, NY, USA) and stored at −70 °C until use in the experiment. To analyze the mRNA template concentration, PCR was performed on a 7500 Real-time PCR system (Applied Biosystems, Foster City, CA, USA) using SYBR^®^ Premix Ex Taq^TM^ (Takara Bio Inc., Shiga, Japan) based on the manufacturer’s instructions and valuable pieces of literature. The data were expressed as the ratio of target mRNA expression to 36B4 (vehicle) mRNA expression. The primers used in this study are summarized in Table S4. These experiments were performed in triplicate and independently duplicated at least three times.

### 3.9. Statistical Analysis

All statistical analysis was performed using GraphPad Prism (version 8.02, GraphPad Software Inc., La Jolla, CA, USA). * *p*-value < 0.05, and ** *p*-value < 0.01 were considered statistically significant difference. RSM was performed to predict the best extract conditions, which was performed using Design-Expert software (version 12, Sta-Ease Inc., Minneapolis, MN, USA).

## 4. Conclusions

In this study, the antiwrinkle activity of enriched Isatidis Folium extract was investigated, the result demonstrated that the enriched Isatidis Folium extract significantly increased the mRNA expression of procollagen and IL-4, and also decreased the mRNA expression of MMP-1, MMP-3, and pro-inflammatory cytokines IL-1β. Furthermore, the major chemical constituents of the enriched Isatidis Folium extract were analyzed using HPLC-PDA-ESI-MS/MS, and five components were conditionally identified. The representative bioactive component TMCA was then selected as a marker compound to control the quality of different samples from the enriched Isatidis Folium extract powder to the finished antiwrinkle cosmetic products. This reliable HPLC-UV method was shown to have good linearity, accuracy, precision, repeatability, and recovery. RSM was conducted to optimize the sample preparation process for TMCA extraction from novel antiwrinkle cream products. The optimal parameters were obtained as follows: sonication time of 38.31 min and the methanol-to-material ratio of 6.47.

## Figures and Tables

**Table 1:** Identification of major compounds in enriched Isatidis Folium extracts by HPLC-PDA-ESI-MS/MS.

No.	Rt ^a^ (min)	UV λ (nm)	Precursorion (*m*/*z*)	Production (*m*/*z*)	Molecular Formula	Identification
1	40.19	213, 269, 336	431.05 (M-H)^−^	311.12 (M-H-C_4_H_9_O_4_)^−^	C_21_H_20_O_10_	isovitexin
2	40.82	213, 269, 338	592.65 (M-H)^−^	311.12 (M-Glc^b^-C_4_H_9_O_4-_H)^−^ 431.00 (M-Glc-H)^−^	C_27_H_30_O_15_	isovitexin-3″-O-glucopyranoside
3	41.78	209, 270, 346	461.20 (M-H)^−^	341.28 (M-H-C_4_H_9_O_4_)^−^	C_22_H_22_O_11_	isoscoparin
4	42.35	208, 270, 348	624.03 (M-H)^−^	341.28 (M-Glc-C_4_H_9_O_4_-H)^−^ 461.12 (M-Glc–H)^−^	C_28_H_32_O_16_	isoscoparin-3″-O-glucopyranoside
5	62.43	229,302	239.00 (M-H)^+^	239.00 (M + H)^+^	C_12_H_14_O_5_	TMCA^c^

^a^ Rt: retention time. ^b^ Glc: beta-D-glucopyranosyl. ^c^ TMCA: 3,4,5-trimethoxycinnamic acid.

**Table 2:** Central composite design matrix of two independent variables in coded (in brackets) and the content of 3,4,5-trimethoxycinnamic acid in the developed cream products.

Run	A: Sonication Time (min)	B: Methanol-to-Material Ratio (mL/g)	Content of TMCA Y (μg/g)
1	40 (0)	7 (0)	30.2
2	40 (0)	4 (−1)	27.3
3	40 (0)	7 (0)	29.7
4	40 (0)	7 (0)	29.6
5	40 (0)	10 (1)	25.2
6	50 (+1)	10 (+1)	21.3
7	40 (0)	7 (0)	29.4
8	50 (+1)	4 (−1)	20.8
9	40 (0)	7 (0)	29.7
10	30 (−1)	10 (+1)	21.9
11	50 (1)	7(0)	24.2
12	30 (−1)	4 (−1)	25.8
13	30 (−1)	7 (0)	26.0

**Table 3:** TMCA concentrations were found in different batches of the samples.

Sample	Batch Number	Concentration
The powder of enriched Isatidis Folium extract	B-1	126.32 ± 0.32 mg/g
	B-2	127.45 ± 0.21 mg/g
	B-3	126.61 ± 0.01 mg/g
Anti-wrinkle cream products	C-1	29.2 ± 0.01 μg/g
	C-2	28.9 ± 0.02 μg/g
	C-3	29.6 ± 0.01 μg/g

## Supplementary Materials

The following are available online at https://www.mdpi.com/2223-7747/9/11/1586/s1, Figures S1–S5: MS and MS/MS spectrum of identified components from enriched Isatidis Folium extract, Figure S6. The plot of actual values versus predicted values for extraction TMCA (**a**) and residual plot (**b**), Table S1. Validation data of TMCA, Table S2. Evaluation of CCD predicted model by ANOVA, Table S3: The response value of prediction and experiment acquired by the optimal conditions, Table S4: Primers sequences using for RT-PCR.
