# Supplementary material for: Evaluation of the Antiwrinkle Activity of Enriched Isatidis Folium Extract and an HPLC–UV Method for the Quality Control of Its Cream Products
Source: Plants (Basel). 2020 Nov 16;9(11):1586. doi: 10.3390/plants9111586 (PMC7698272; doi:10.3390/plants9111586)
Supplement: Supplementary file 1 [file plants-09-01586-s001.pdf]

SUPPLEMENTARY MATERIAL

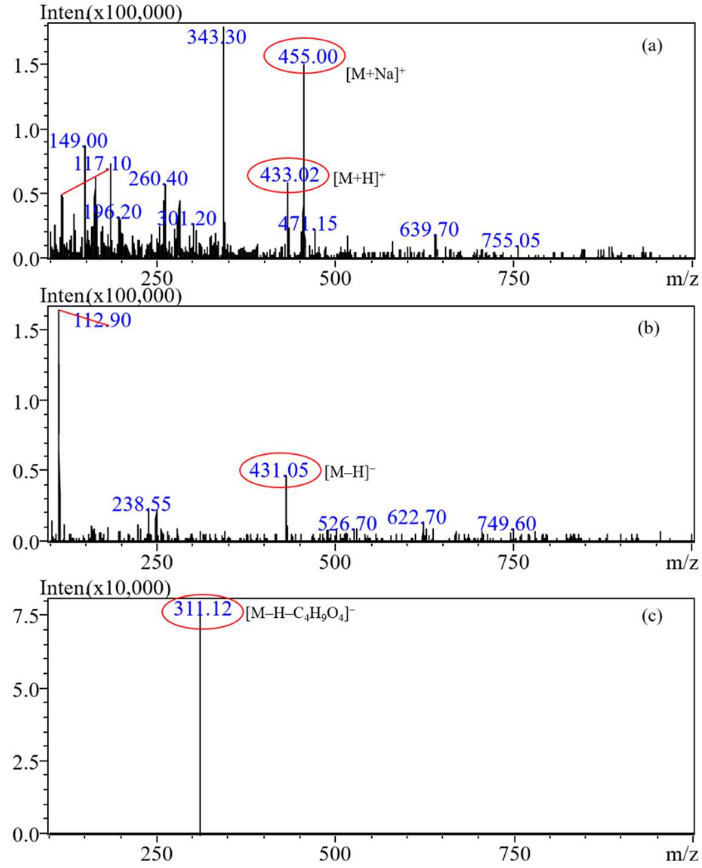

Figure S1. Mass spectrometry (MS) and MS/MS spectrum of isovitexin. (**a**) MS spectrum in positive mode; (**b**) MS spectrum in negative mode; (**c**) MS/MS spectrum in negative mode

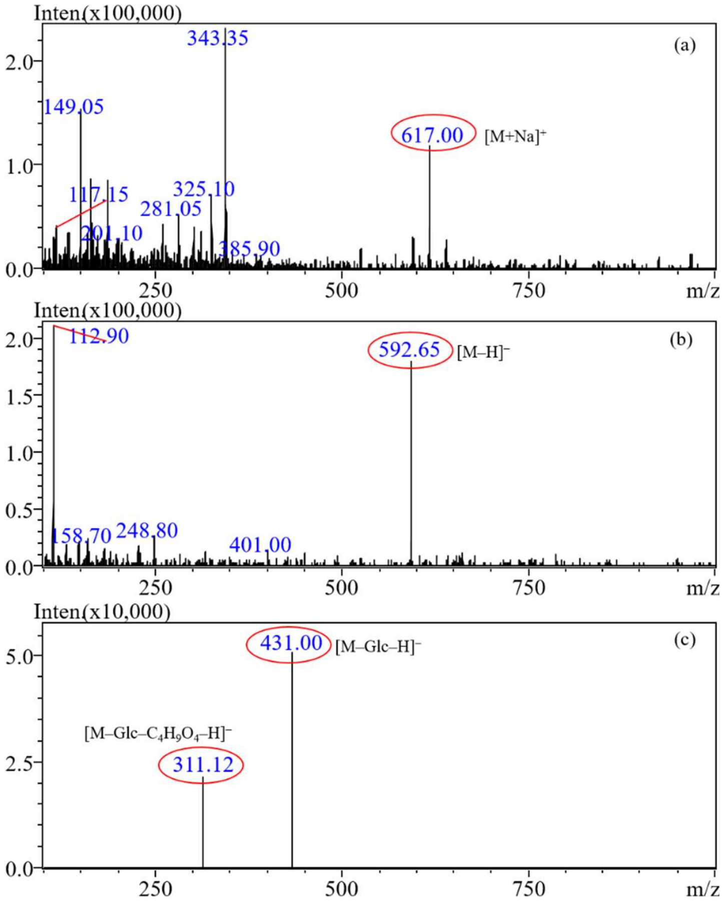

Figure S2. Mass spectrometry (MS) and MS/MS spectrum of isovitexin-3″-O-glucopyranoside. (**a**) MS spectrum in positive mode; (**b**) MS spectrum in negative mode; (**c**) MS/MS spectrum in negative mode.

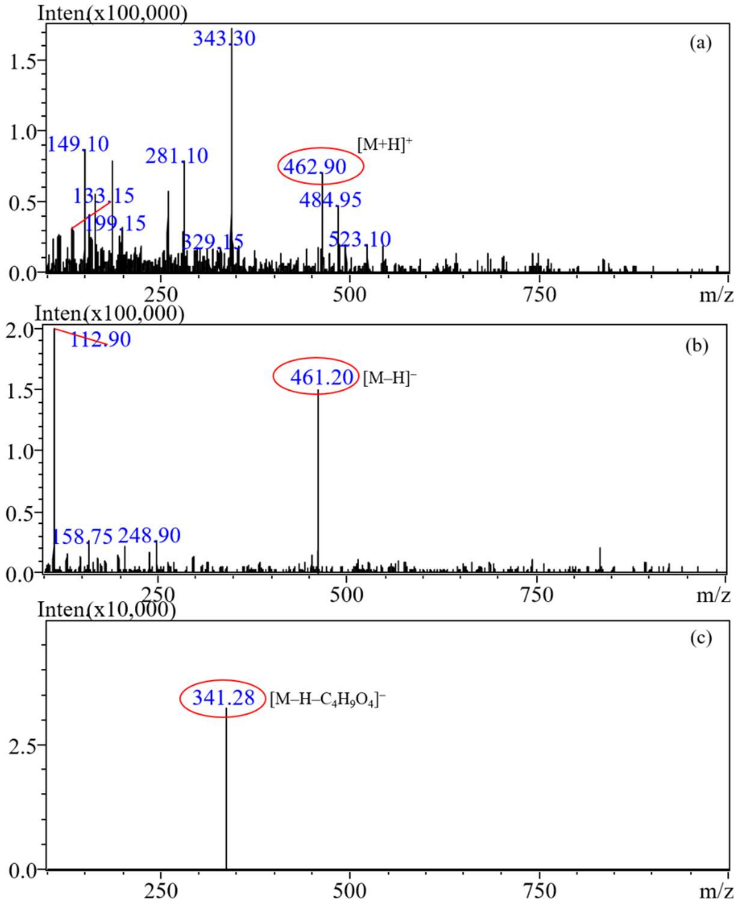

Figure S3. Mass spectrometry (MS) and MS/MS spectrum of isoscoparin. (**a**) MS spectrum in positive mode; (**b**) MS spectrum in negative mode; (**c**) MS/MS spectrum in negative mode.

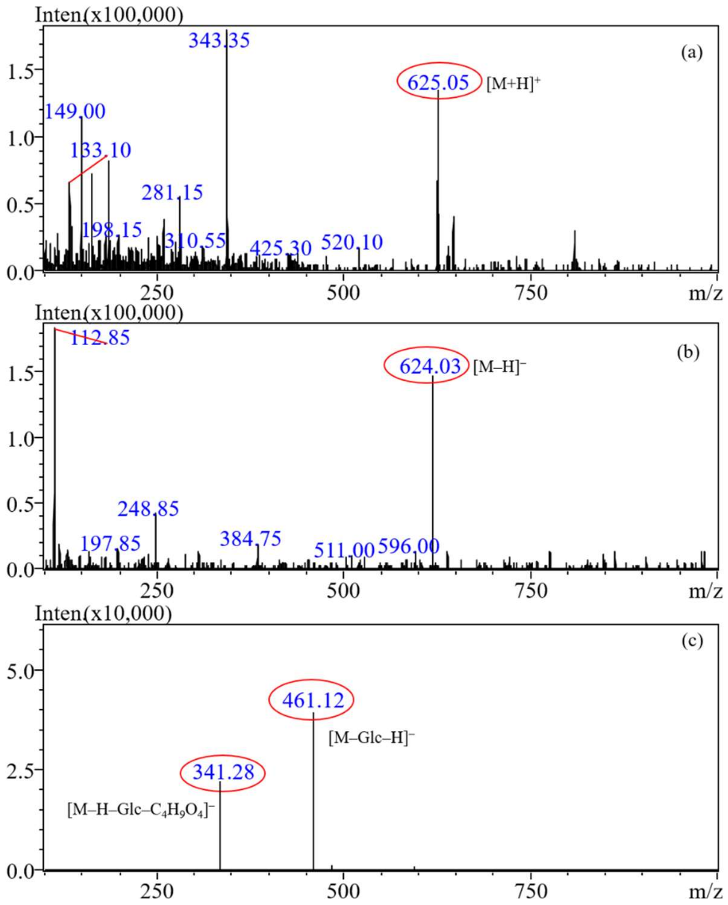

Figure S4. MS and MS/MS spectrum of isoscoparin-3″-O-glucopyranoside. (**a**) MS spectrum in positive mode; (**b**) MS spectrum in negative mode; (**c**) MS/MS spectrum in negative mode.

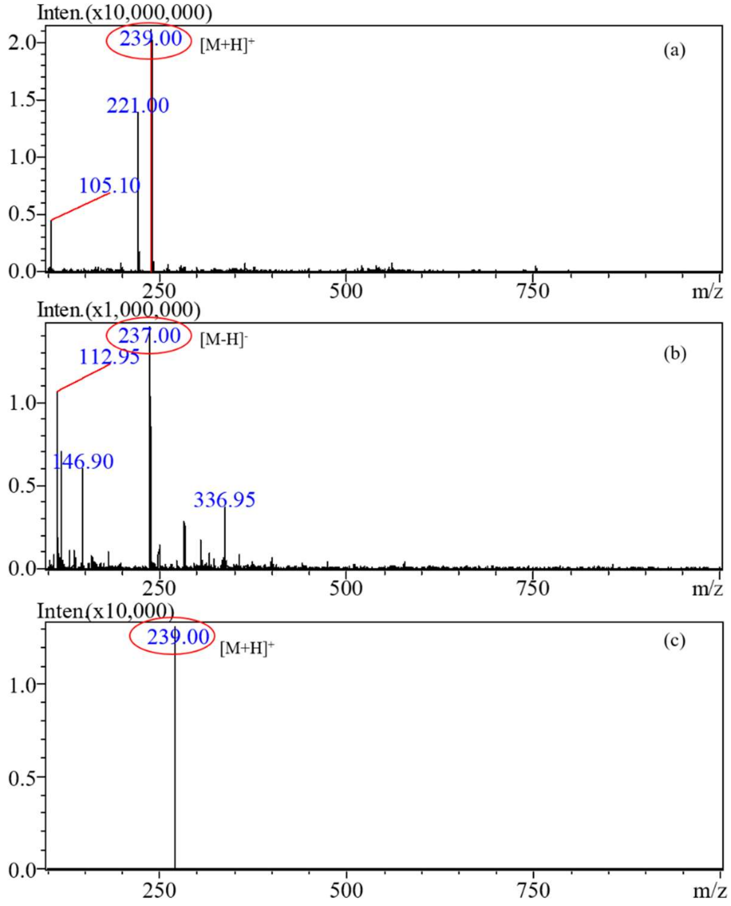

Figure S5. Mass spectrometry (MS) and MS/MS spectrum of TMCA (3,4,5-trimethoxycinnamic acid). (**a**) MS spectrum in positive mode; (**b**) MS spectrum in negative mode; (**c**) MS/MS spectrum in positive mode.

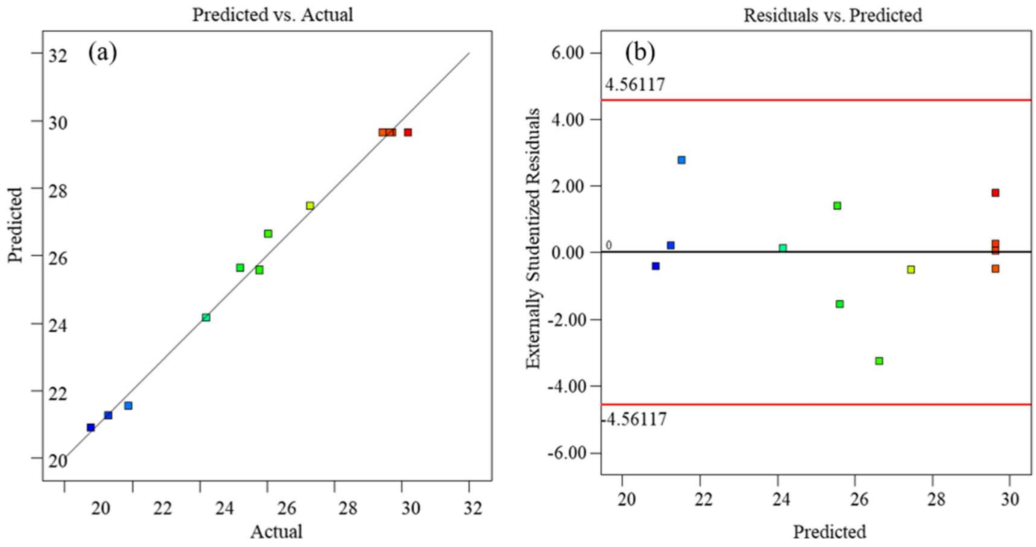

Figure S6. The plot of actual values versus predicted values for extraction 3,4,5-trimethoxycinnamic acid (**a**) and residual plot (**b**).

Table S1. Validation data of TMCA (3,4,5-trimethoxycinnamic acid).

Parameters	TMCA
Linearity range (μg/mL)	1–10
Coefficient of determination (R^2)	0.9996
Equation	y = 360x–60
limit of detection (ng/mL)	6.08
limit of quantitation (ng/mL)	18.44
Precision	
Intra-day (%RSD)	0.2–0.6
Inter-day (%RSD)	1.9–3.2
Accuracy	
Intra-day (%RSD)	97.3–101.4
Inter-day (%RSD)	103.1–107.0
Recovery (%)	99.18–107.20
Repeatability	
Retention time (%RSD)	0.23
Content (%RSD)	0.18

[a]RSD: relative standard deviation

Table S2. Evaluation of central composite design (CCD) predicted model by ANOVA.

Source	SS[a]	df[b]	Mean Square	F-value[c]	*p*-Value	
Model	139.99	5	28	175.99	< 0.0001	significant
A-Sonication time	9.23	1	9.23	57.99	0.0001	
B-Methanol-to-material ratio	4.99	1	4.99	31.35	0.0008	
AB	4.84	1	4.84	30.42	0.0009	
A^2	49.51	1	49.51	311.22	< 0.0001	
B^2	26.35	1	26.35	165.65	< 0.0001	
Residual	1.11	7	0.1591			
Lack of Fit	0.8053	3	0.2684	3.48	0.1297	Not significant
Pure Error	0.3083	4	0.0771			
Cor Total[d]	141.10	12				
R^2	0.9921					
C.V. %	1.52		Adjusted R^2	0.9865		
			Predicted R^2		0.9495	

[a] SS: Sum of squares. [b] df: Degree of freedom. [c] *F* value was calculated by mean square of residual divided by mean square of source. [d] Cor total: Sum of squares and df.

Table S3. The response value of prediction and experiment acquired by the optimal conditions (*n* = 3).

	A (min)	B: (mL/g)	Content of TMCA[a] Y (μg/g)
Predicted	38.31	6.47	29.81
Experimental	38	6	29.17
Matching (%)			97.85%

[a]TMCA: 3,4,5-trimethoxycinnamic acid

Table S4. Primers sequences using for real-time reverse-transcription polymerase chain reaction.

Primer		Sequence
Procollagen	Forward	3′-CTC CAA CGA GAT CGA GTA C-5′
	Reverse	3′-GTT ACA GGA AGC AGA CAG G-5′
MMP-1	Forward	3′-GAT GTG GAG TGC CTG ATG TG-5′
	Reverse	3′-TGC TTG ACC CTC AGA GAC CT-5′
MMP-3	Forward	5′-TAG GAG GTT ATC CTA AAA GCA-3′
	Reverse	5′-CCA GCT ATT GCT CTT CAA GCA-3′
IL-1β	Forward	5′-GAC TCA TGG GAT GAT GAT GAT AAC-3′
	Reverse	5′-CCA TAC TTT AGG AAG ACA CGG ATT-3′
IL-4	Forward	5′-TCG GCA TTT TGA ACG AGG TC-3′
	Reverse	5′-GAA AAG CCC GAA AGA G TC-3′
36B4	Forward	5′-TGG GCT CCA AGC AGA TGC-3′
	Reverse	5′-GGC TTC GCT GGC TCC CAC -3′
